# Overexpression of an evolutionarily conserved drought-responsive sugarcane gene enhances salinity and drought resilience

**DOI:** 10.1093/aob/mcz044

**Published:** 2019-05-24

**Authors:** Kevin Begcy, Eduardo D Mariano, Carolina G Lembke, Sonia Marli Zingaretti, Glaucia M Souza, Pedro Araújo, Marcelo Menossi

**Affiliations:** 1 Laboratório de Genoma Funcional, Departamento de Genética, Evolução, Microbiologia e Imunologia, Instituto de Biologia, Universidade Estadual de Campinas, Campinas, Brazil; 2 Laboratório de Transdução de Sinal, Departamento de Bioquímica, Instituto de Química, Universidade de São Paulo, São Paulo, Brazil; 3 Unidade de Biotecnologia, Universidade de Ribeirão Preto, Ribeirão Preto, São Paulo, Brazil; 4 Cell Biology and Plant Biochemistry, Biochemie-Zentrum Regensburg, University of Regensburg, Regensburg, Germany

**Keywords:** Abiotic stresses, drought, salinity, tolerance, *Saccharum officinarum*, gas exchange parameters, transgenic tobacco

## Abstract

**Background and Aims:**

Improving drought adaptation is more pressing for crops such as sugarcane, rice, wheat and maize, given the high dependence of these crops on irrigation. One option for enhancing adaptation to water limitation in plants is by transgenic approaches. An increasing number of genes that are associated with mechanisms used by plants to cope with water scarcity have been discovered. Genes encoding proteins with unknown functions comprise a relevant fraction of the genes that are modulated by drought. We characterized a gene in response to environmental stresses to gain insight into the unknown fraction of the sugarcane genome. *Scdr2* (Sugarcane drought-responsive 2) encodes a small protein and shares highly conserved sequences within monocots, dicots, algae and fungi.

**Methods:**

Plants overexpressing the *Scdr2* sugarcane gene were examined in response to salinity and drought. Measurements of the gas exchange parameters, germination rate, water content, dry mass and oxidative damage were performed. Seeds as well as juvenile plants were used to explore the resilience level of the transgenic plants when compared with wild-type plants.

**Key Results:**

Overexpression of *Scdr2* enhanced germination rates in tobacco seeds under drought and salinity conditions. Juvenile transgenic plants overexpressing *Scdr2* and subjected to drought and salinity stresses showed higher photosynthesis levels, internal CO_2_ concentration and stomatal conductance, reduced accumulation of hydrogen peroxide in the leaves, no penalty for photosystem II and faster recovery after submission to both stress conditions. Respiration was not strongly affected by both stresses in the *Scdr2* transgenic plants, whereas wild-type plants exhibited increased respiration rates.

**Conclusions:**

*Scdr2* is involved in the response mechanism to abiotic stresses. Higher levels of *Scdr2* enhanced resilience to salinity and drought, and this protection correlated with reduced oxidative damage. *Scdr2* confers, at the physiological level, advantages to climate limitations. Therefore, *Scdr2* is a potential target for improving sugarcane resilience to abiotic stress.

## INTRODUCTION

Abiotic stresses, such as drought and salinity, are major limitations for crop productivity resulting in significant yield losses annually (Begcy and Dresselhaus, 2018). Maintaining plant productivity in less favourable environments is a continuous challenge for plant breeders. As agriculture is gradually pushed to marginal lands and the freshwater supply fluctuates due to erratic weather patterns, drought continues to challenge our ability to meet the needs of an increasing world population. In several regions, irrigation produces as a side effect soil salinization due to the use of poor-quality water (Parihar *et al.*, 2015). Sugarcane, the primary sugar-producing crop, which has recently been successfully used as a source of clean energy (Dal-Bianco *et al.*, 2012; Hoang *et al.*, 2017), is also affected by both drought and salinity (Vasantha *et al.*, 2010; Ferreira *et al.*, 2017). Owing to the growing demand for food and renewable energy sources, sugarcane cultivation has expanded to regions with lower water supplies and higher temperatures, where drought is quite common. Moreover, in certain regions, irrigation has caused soil salinization, which also interferes with sugarcane productivity. Therefore, the development of new cultivars with an increased tolerance to drought and salinity stresses is key to meet the increasing demand for sugar and bioethanol.

The wide array of mechanisms that plants use to maintain yield production in different stressful environments and the low heritability of such traits complicate the process of producing improved cultivars. Nevertheless, our understanding of plant responses to stress has significantly improved with advances in related areas, such as genetics, physiology and molecular biology (Gao *et al.*, 2007; Perez-Clemente *et al.*, 2013). There is increasing evidence suggesting the potential role of several genes in reducing the limitations imposed on crops by environmental stresses (Umezawa *et al.*, 2006; Begcy *et al.*, 2012; Fujita *et al.*, 2013; Begcy and Walia, 2015; Li *et al.*, 2015; Chen *et al.*, 2016). Genes encoding proteins with unknown functions are interesting resources in discovering new plant features (Luhua *et al.*, 2013). In most genome projects, unknown genes represent the largest category of proteins: 30 % in rice (Yu *et al.*, 2002), 33 % in maize (Schnable *et al.*, 2009) and 40 % in soybean (Schmutz *et al.*, 2010). In sugarcane, 237 954 ESTs (expressed sequence tags) were obtained from 27 sugarcane cDNA libraries from multiple tissues of four cultivars that were exposed to a variety of conditions, such as low and high temperature and endophytic nitrogen-fixing bacterial interactions, among others, during different developmental stages. The EST collection was assembled into 43 141 putative and unique sugarcane transcripts (Vettore *et al.*, 2003), of which 38 % encoded proteins with unknown functions (Vettore *et al.*, 2001).

In a previous study, we identified 179 differentially expressed genes when sugarcane plants were exposed to a variety of biotic and abiotic stresses (Rocha *et al.*, 2007). Interestingly, drought caused the most significant changes in the sugarcane transcriptome, affecting the expression of 93 genes. Among these genes, several encoded proteins with unknown functions, and one of them, *Scdr1* (*S*ugar*c*ane *d*rought-*r*elated-1), has been shown to enhance tolerance against environmental stresses (Begcy *et al.*, 2012). Here, we have functionally characterized the role of *Scdr2*, a small drought-induced protein-encoding gene. The *Scdr2* transcriptional expression pattern was evaluated in four sugarcane cultivars with contrasting drought tolerance when exposed to drought. Transgenic tobacco plants overexpressing *Scdr2* displayed higher photosynthesis levels, stomatal conductance, transpiration rates and internal CO_2_ concentrations than wild-type (WT) plants when exposed to drought and salinity. Additionally, under these conditions, the transgenic *Scdr2* plants presented higher biomass. Our data indicate that *Scdr2* is a potential target for improving sugarcane resilience to abiotic stress.

## MATERIALS AND METHODS

### Plant material and growth conditions

Sugarcane (*Saccharum officinarum*) plants of two cultivars with higher drought tolerance (SP83-5073 and SP83-2847) and two with lower drought tolerance (SP90-1638 and SP86-155) were grown in controlled conditions as described by Rocha *et al.* (2007). Tobacco (*Nicotiana tabacum*, var. SR1) seedlings were obtained and grown in 325 mL pots containing four parts (v/v) of peat (Bacto, Michigan Peat Co., Houston, TX, USA) and one part of sand as described by Begcy *et al.* (2012). Plants with 10–12 leaves were then transferred to 1 L pots with the same solid substrate. The plants were grown with a 16/8 h light/dark cycle (300–400 μmol m^–2^ s^–1^) at 25 °C and a relative humidity of 75–80 % until flowering to produce seeds, or for 5 weeks to be used in drought and salinity experiments.

### Real-time qPCR and semi-quantitative RT–PCR

Quantitative real-time PCR was performed using gene-specific primers as previously described by Begcy and Dresselhaus (2017). Sugarcane leaves from the SP90-1638 and SP86-155 (low tolerance to drought) and SP83-5073 and SP83-2847 (high tolerance to drought) cultivars were collected after 24, 72 and 120 h from the beginning of the suspension of water supply. RNA from sugarcane leaves was extracted using Trizol (Invitrogen, USA) according to the manufacturer’s instructions. First-strand cDNA was produced from 2 μg of total RNA using oligo d(T)18 primers and the Superscript III Kit according to the manufacturer’s instructions (Life Technologies, USA). The 2^–ΔΔCt^ method (Livak and Schmittgen, 2001) was used to calculate the relative expression of genes (experimental/control) using a sample that was not subjected to stress as a control and a gene encoding polyubiquitin as a reference. In each treatment, leaves from six plants were used for each time point, and the statistical analysis was performed as described by Rocha *et al.* (2007). When P was ≥0.95, the profile expression was considered statistically significant. For semi-quantitative reverse transcription–PCR (RT–PCR), total RNA from leaves of WT and transgenic tobacco plants was extracted using the RNeasy Plant Mini Kit (Qiagen, USA); the amplification of *Scdr2* was as follows: 1 min at 94 °C, followed by 21 cycles of 30 s at 94 °C, 60 s at 58 °C and 75 s at 72 °C, and 1 μL of cDNA was used as a template. As control, the tobacco actin gene (AB158612.1) was amplified under the same PCR conditions, except that the number of PCR cycles was increased to 28 and the annealing temperature was set to 60 °C. The relative densitometric ratios were determined using ImageJ (http://rsbweb.nih.gov/ij/), and the experiment was repeated three times.

### Construction of plant expression vectors and transformation of tobacco plants

The full-length clone of *Scdr2* was obtained from the Brazilian Clone Collection Center (BCCCENTER, Brazil). The complete coding sequence (accession number JX094282) was amplified by PCR using gene-specific primers (forward, 5′ GACTCCTCTCTCTTCTCTCGC 3′; and reverse, 5′ CATATGCTGCTGCTCTGC 3′) and then cloned into pGEMT-Easy (Promega, USA). A *Bam*HI*/Xba*I fragment was cloned into the pRT104 vector and digested with the same enzymes. The expression cassette comprising the 35S promoter, the *Scdr2* coding sequence and the NOS terminator was transferred as a *Hin*dIII fragment to the pCAMBIA 2301 vector (Cambia, Australia) and digested with the same enzymes. The resulting construct was named pCAMBIA2301::*Scdr2* and was introduced into *Agrobacterium tumefaciens* (strain GV3101, Clontech, USA).

For transformation of tobacco with *A. tumefaciens*, young leaves were used, as described by Begcy *et al.* (2012). Once the young plantlets developed roots, they were transferred to a solid substrate (Plantmax HT, Eucatex, Brazil) and grown in a growth chamber under the previously described conditions. Homozygous transgenic seeds from the T_3_ generation were used throughout the experiment.

### Drought and salinity stress treatments

Wild-type and transgenic tobacco seeds were surface-sterilized with 70 % (v/v) ethanol for 1 min and were then kept in 2 % (v/v) NaClO for 30 min and washed in sterile distilled water 5–6 times. Thirty seeds per plate were sown onto Petri dishes containing Murashige and Skoog (MS) medium and were maintained in a growth chamber at 23 °C with a 16/8 h light/dark cycle (300–400 μmol m^–2^ s^–1^). Four independent experiments were performed using a randomized complete block design (RCBD). To simulate drought and salinity stress, different concentrations of mannitol (0, 200 and 300 mm) and NaCl (0, 100 and 175 mm) were used, respectively. Germination was scored daily for 15 d and seeds were considered as germinated when the radicle was at least 2 mm long. Graphics were plotted as the cumulative percentage of seeds that have germinated by the end of the 15 d of the experiment.

For the stress imposition on transgenic plants, plants were irrigated daily with 70 mL of water for 5 weeks before initiating the stress treatments. For salinity stress, seedlings were irrigated with 70 mL of 100 mm NaCl, and for drought stress, with 70 mL of 200 mm mannitol, for 10 d, after which the seedlings were allowed to recover by irrigation with regular water for 3 d, as described by Begcy *et al.* (2012). The experimental design was an RCBD with four replications. Each replication was conducted four times with *n* = 5 plants per genotype.

### Physiological measurements of the transgenic plants and hydrogen peroxide determination

The uppermost completely expanded leaf (the fourth counting from the bottom to the top) of each genotype was used to measure stomatal conductance (*g*_s_), transpiration rate (*E*) and net photosynthetic rate (*A*) using an infrared gas analyser (IRGA -LCpro+; ADC BioScientific, UK) at a CO_2_ concentration of 360 μL L^–1^, a saturating light intensity of 1000 μmol m^–2^ s^–1^ and a gas flow rate of 200 mL min^–1^. The temperature inside the leaf chamber was maintained at 25 °C. Fluorescence in the form of the *F*_v_/*F*_m_ ratio was measured using a mini-pulse fluorometer on the same leaves used previously (MiniPAM, Walz, Germany).

Plant shoots were weighed to obtain the fresh weight (f. wt) and then dried in an oven at 80 °C for 72 h to assess their dry weight (d. wt), to evaluate their water content and biomass accumulation. The leaf water content was calculated as (f. wt − d. wt)/(f. wt × 100). To measure the leaf respiration, 5-week-old plants were grown in a growth chamber at 25 °C with a 16/8 h light/dark period. To avoid transient metabolic activity following darkening, the night respiration was measured after 3 h of acclimation to darkness. The IRGA (infrared gas analyser) was also used to measure carbon dioxide production as described previously (Begcy *et al.*, 2011; Mattiello *et al.*, 2014; Begcy *et al.*, 2018). To quantify H_2_O_2_, a modified ferrous ammonium sulphate/xylenol orange (FOX) method was used as described by Begcy *et al*. (2011, 2012). We produced a standard curve of known concentrations of H_2_O_2_ (0, 2.5, 5, 7.5, 10, 12.5 and 15 μm), and this curve was used in the quantification of H_2_O_2_ in each sample.

### Statistical analysis and identification of ScDR2 homologous proteins

The sequence of ScDR2 protein from sugarcane was used for searching homologous sequences in the NCBI protein database, and sequences with similarities >50 % were used for further analysis. Bootstrap analysis was performed with 1000 replicates to assess the level of statistical support for each tree node.

Statistical analyses were performed using the R software/environment. To access the statistical significance of expression ratios, we assumed a log-normal model and calculated the probability *P* (sample > reference) and *P* (sample < reference) for up- and downregulated genes, respectively. The expression profile was considered validated when *P* was ≥ 0.95. Data from four independent RCBD experiments, where each experiment had *n* = 5 per genotype, were used. Data were expressed as mean, median, minimum value (min) and maximum value (max), and a *P*-value of 0.05 was used as the significance level.

## RESULTS

On average, about 30 % of all plant genomes encode proteins of unknown function (Yu *et al.*, 2002; Schnable *et al.*, 2009; Schmutz *et al.*, 2010). This particular group of proteins is characterized by the lack of defined domains or motifs (Luhua *et al.*, 2013). Transcriptional and proteome profiling under environmental conditions indicate their putative role in stress mechanisms. To elucidate the role of proteins with unknown function, which are involved in abiotic stress responses, we functionally characterized the role of *Scdr2* in response to abiotic stimuli.

### *Scdr2* displayed a variable transcriptional expression in several sugarcane drought-stressed cultivars

**Fig. 1:**
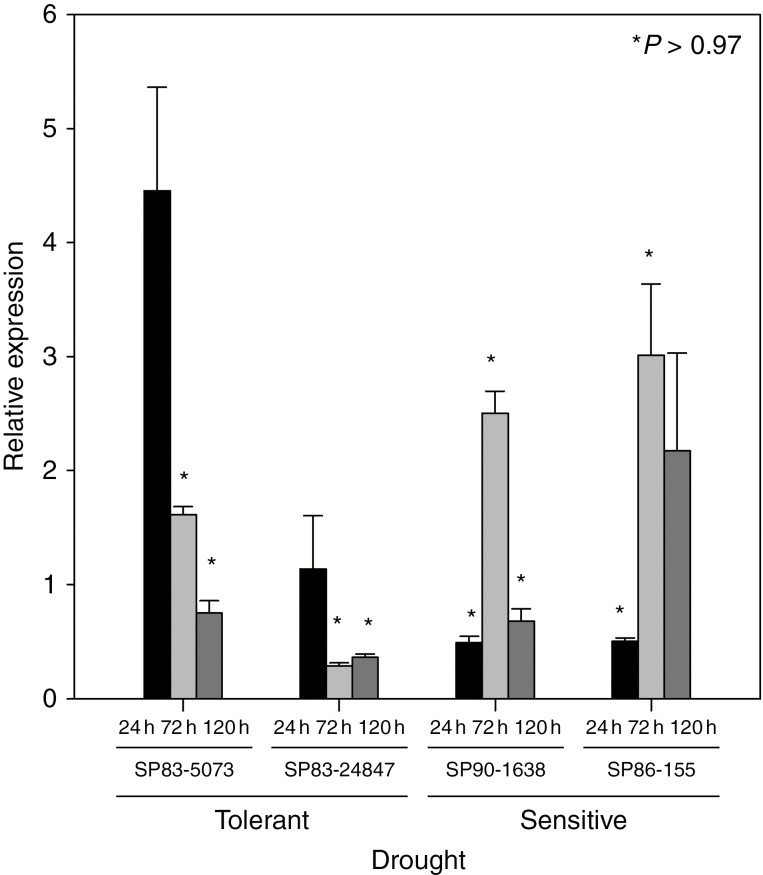
*Scdr2* gene expression profile in drought-stressed sugarcane. *Scdr2* gene expression was evaluated in four sugarcane cultivars (SP83-5073, SP83-2847, SP90-1638 and SP86-155) after 24, 72 and 120 h of control or drought stress conditions. A gene encoding polyubiquitin was used as the reference gene for normalization. Treatments that produced a statistically significant difference in gene expression are marked with an asterisk. *Scdr2* relative expression was normalized to the control condition (*n* = 6).

In a previous study, we evaluated transcriptional responses of sugarcane to several abiotic and biotic stresses including drought, phosphate starvation, herbivore attack, methyl jasmonate and abscisic acid (ABA) treatment, and exposure to two N_2_-fixing endophytic bacteria (Rocha *et al.*, 2007). Particularly during drought stress, we identified a group of 93 genes that were differentially expressed, of which about 14 % of them encoded proteins with unknown functions. To explore further whether this particular set of genes plays a role during environmental stresses, we selected one of them for functional analysis. The sugarcane assembled sequence SCRFLR2038D12.g encoding a small protein (77 amino acids) was named *Scdr2* [*S*ugar*c*ane *d*rought-*r*elated 2; the first sugarcane gene encoding an unknown protein related to drought, *Scdr1*, was described by Begcy *et al.* (2012)]. First, we evaluated the expression pattern of *Scdr2* in leaves of several sugarcane cultivars with contrasting tolerance to drought; SP83-5073 and SP83-2847 (cultivars with higher tolerance), and SP86-155 and SP90-1638 (cultivars with lower tolerance). SP83-5073 was used in the microarray experiments from Rocha *et al.* (2007). *Scdr2* transcriptional expression was measured after 24, 72 and 120 h of water deprivation (Fig. 1). No clear correlation was observed among all analysed cultivars. They displayed distinct patterns of *Scdr2* expression at the three time points evaluated. SP83-5073 showed the highest induction of *Scdr2* in response to drought (4-fold) after 24 h, and the expression decreased at later time points. SP83-2847 responded in a rather distinct way; there was no induction of *Scdr2* after 24 h and its expression was repressed after 72 and 120 h. SP90-1638 had a 2.7-fold higher *Scdr2* expression after 72 h, while the expression was repressed in response to drought at the other time points. *Scdr2* expression was repressed by drought after 24 h in SP86-155, while a 2- to 3-fold induction of expression was observed after 72 and 120 h. These data indicate that the regulation of *Scdr2* expression diverged among the different sugarcane cultivars. Interestingly, the *Scdr2* homologue in maize was also induced after moderate and severe drought stress (Zheng *et al.*, 2010) (Supplementary Data Fig. S1), indicating that this gene might be involved in drought responses in other species.

### *ScDR2* is conserved in plants, algae and fungi

The *Scdr2* transcript sequence (GenBank accession number AFY12046) and the deduced amino acid sequence are shown in Supplementary Data Fig. S2. *Scdr2* comprises 234 nucleotides and encodes a protein with 77 amino acids. A high degree of similarity was observed when ScDR2 was aligned with sequences from monocots, dicots, algae and fungi (Supplementary Data Fig. S3A), and maize (95 %), sorghum (96 %) and rice (88 %) homologues showed the highest similarity. Similarly, ScDR2 also displayed a high degree of similarity with proteins from dicotyledonous species, varying from 61 to 87 % in arabidopsis and *Vitis vinifera*, respectively. Interestingly, this protein is conserved even in fungi (54 % similarity), suggesting a possible conserved function. Overall, a high degree of similarity was observed in all species that were analysed. A Neighbor–Joining tree was constructed based on the highly conserved alignment (Supplementary Data Fig. S3B). ScDR2 was grouped into the same clade as the analysed monocotyledonous plants, while the dicot plants were grouped into two different clades (Supplementary Data Fig. S3A). The high degree of similarity between the dicotyledonous and monocotyledonous proteins prompted us to evaluate the role of *Scdr2* using transgenic tobacco plants.

### Heterologous expression of *Scdr2* in tobacco plants

**Fig. 2:**
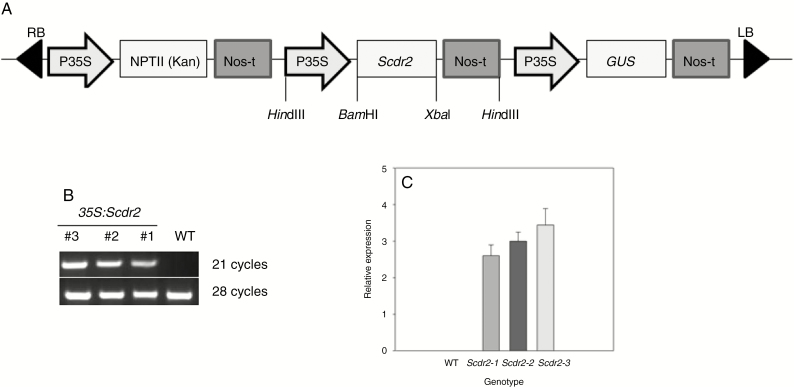
Cloning strategy used for *Scdr2* overexpression in tobacco plants. (A) The complete coding sequence of *Scdr2* was cloned under the control of the constitutive *Cauliflower mosaic virus* (CaMV) 35S promoter (p35S) and with the NOS polyadenylation signal (Nos-t) using pCambia2301 as the backbone. *nptII* (kanamycin resistance) gene expression was also driven by the p35S promoter. LB and RB correspond to the T-DNA left and right borders, respectively. Positions of some of the restriction sites are indicated. (B) Expression of *Scdr2* in three independent T_3_ generation transgenic lines and the WT. Total RNA was extracted from 2-week-old seedlings and then analysed using semi-quantitative RT–PCR. The *Scdr2* gene product was obtained after 21 cycles, while the product of the tobacco actin gene, which was used as an internal standard, was obtained after 28 cycles (*n* = 3). (C) Densitometric analysis of *Scdr2* expression that was obtained from the semi-quantitative RT–PCR shown in (B).

To characterize *Scdr2* functionally, the gene was overexpressed in tobacco plants. For this purpose, a p35S::Scdr2 expression cassette was inserted into the pCambia2301 vector (Fig. 2A) and used to transform tobacco explants. An initial screening for positive events was performed by scoring the β-glucuronidase (GUS) activity using X-Gluc as a substrate. We identified 12 plants showing strong blue staining. The segregation analysis of these plants allowed the identification of those presenting a 3:1 pattern for kanamycin resistance in the T_2_ generation. To further confirm the integration of *Scdr2*, genomic DNA from transformed plants was used to amplify the sugarcane gene. Several plants carried the transgene (Fig. 2B), in agreement with the X-Gluc staining data; the plants without GUS activity were negative for DNA amplification (data not shown). The progenies of self-pollinated T_2_ plants were screened in MS medium containing kanamycin to identify the homozygous plants that were used in the experiments with the transgenic plants. Three independent T_3_ lines that tested positive for the presence of *Scdr2* by PCR and showed X-Gluc staining were chosen to evaluate the effects of *Scdr2* overexpression. The expression of *Scdr2* in these lines was confirmed by semi-quantitative RT–PCR (Fig. 2B, C).

### *Scdr2*-overexpressing plants showed enhanced seed germination rate under salinity and drought stress

**Fig. 3:**
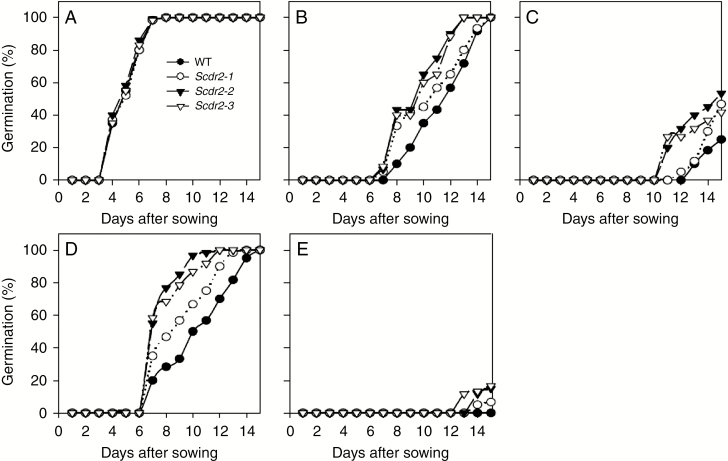
Drought and salinity effect on germination of tobacco seeds. The percentage of germination of three transgenic tobacco lines (*Scdr2*-1 to *Scdr2*-3) and one wild-type tobacco (WT) at different concentrations of mannitol (drought) and NaCl (salinity) were evaluated over a 15 d period. (A) Control; (B) 200 mm mannitol; (C) 300 mm mannitol; (D) 100 mm NaCl; and (E) 175 mm NaCl (n = 5).

Germination is critical for plant fitness in adverse environments. To assess the effect of *Scdr2* on seed germination during drought, seeds from homozygous plants from three independent transformation events were germinated in a culture medium containing mannitol. Cumulative germination of transgenic seeds from *Scdr2*-transformed plants showed 100 % germination after being sown in control medium, similarly to WT seeds (Fig. 3A). In order to test different levels of drought, we sowed both *Scrd2*-overexpressing plants and the WT in media containing distinct mannitol concentrations. Drought imposed by 200 mm mannitol caused a negative effect on both *Scdr2* and WT seeds. However, even though a delay in germination was observed, *Scdr2* seeds performed better than WT seeds, spending less time to achieve 100 % germination (Fig. 3B). When a stronger drought was imposed, by using 300 mm mannitol, cumulative germination of the WT seeds was significantly inhibited (to about 25 %); however, cumulative germination of the transgenic lines seeds was only moderately inhibited (to 40–60 %; Fig. 3C).

Since drought and salinity stress responses show similarities at the physiological and molecular levels (Bartels and Sunkar, 2005), we also evaluated the effect of salinity in seeds overexpressing *Scdr2* (Fig. 3D, E). First, we analysed the cumulative seed germination rate under a moderate level of salinity induction (100 mm NaCl). Both *Scdr2* and WT seeds germinated 100 % after 15 d of stress; however, a faster rate of cumulative germination was noticeable on *Scdr2* transgenic seeds compared with WT seeds (Fig. 3D). Under a severe salinity stress condition (175 mm NaCl), germination of WT seeds was completely inhibited, whereas *Scdr2* seeds were able to maintain about 20 % of germination (Fig. 3E). These data strongly indicate the role of *Scdr2* in improving seed germination under salinity and drought stress conditions.

### *Scdr2* improved physiological responses under drought and salinity stress

**Fig. 4:**
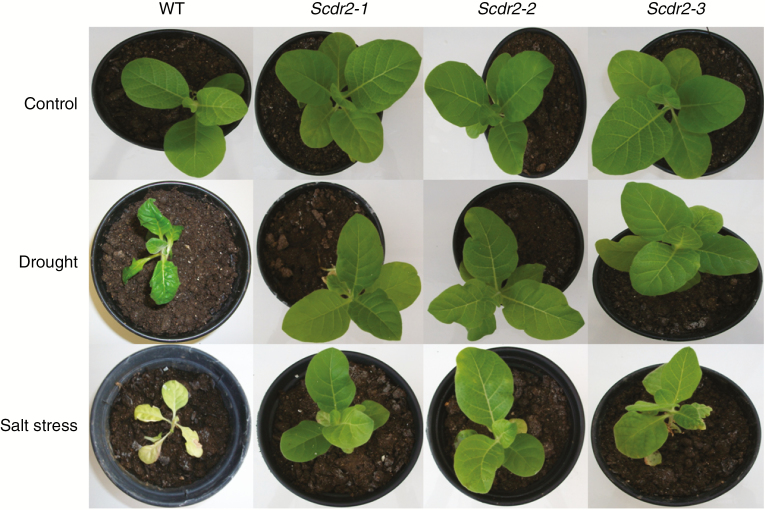
Effect of drought and salinity in tobacco seedlings overexpressing *Scdr2*. First row: the wild type (WT) and three independent transformants overexpressing the *Scdr2* gene that were grown under control conditions for 13 d. Middle row: plants that were irrigated with 200 mm mannitol for 10 d and then irrigated with pure water for 3 d. Bottom row: plants that were irrigated for 10 d with 100 mm NaCl and then irrigated with pure water for 3 d (n = 5).

Under control conditions, the phenotype of *Scdr2* transgenic plants was similar to that of WT plants (Fig. 4). When challenged with mannitol or NaCl for drought and salinity, respectively, WT plants showed clear symptoms of stress, having wilted leaves after the recovery period. Transgenic *Scdr2* plants showed a clear contrast to the WT plants, maintaining a phenotype similar to that of plants grown under control conditions (Fig. 4).

**Fig. 5:**
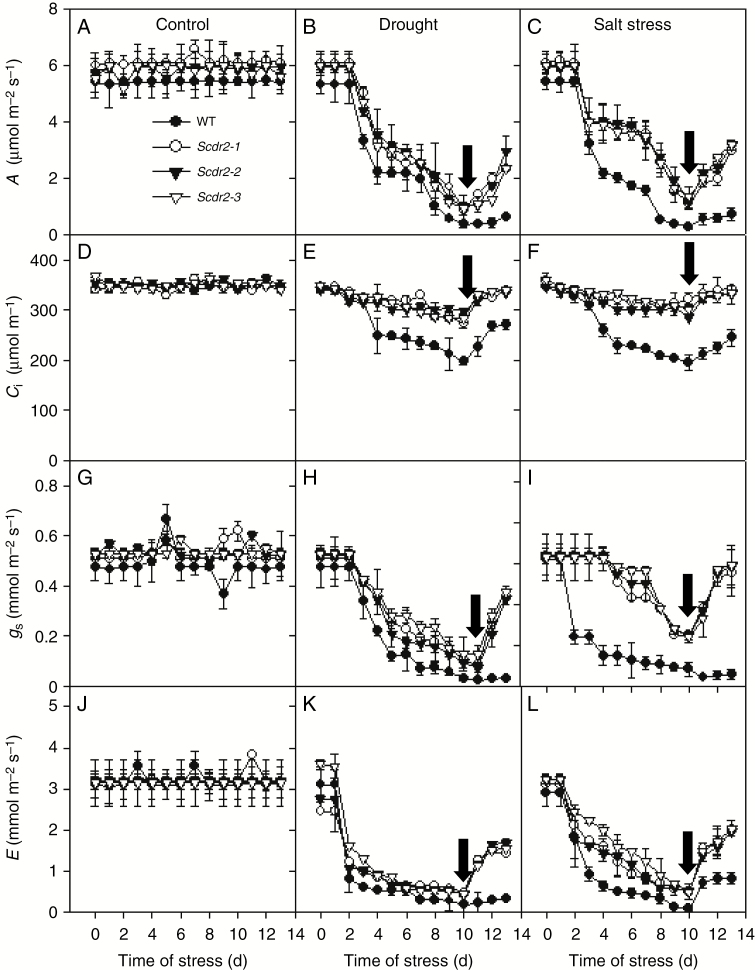
Drought and salinity stress effects on gas exchange parameters in wild-type and transgenic tobacco plants overexpressing *Scdr2*. (A–C) Net photosynthesis (*A*); (D–F) internal leaf CO_2_ concentration (*C*_*i*_); (G–I) stomatal conductance (*g*_s_); (J and K) transpiration rate (*E*). (A), (D), (G) and (J) Control treatment; (B), (E), (H) and (K) 200 mm mannitol (drought); (C), (F), (I) and (L) 100 mm NaCl (salinity stress). Data are presented as the mean ± s.d. from three independent experiments (*n* = 5 in each experiment). Plants were drought and salt stressed for 10 d and then irrigated with pure water for 3 d for the stress recovery period. Arrows indicate the timing of re-watering.

During drought and salinity experiments (Fig. 4), net photosynthesis (*A*), internal carbon concentration (*C*_i_), stomatal conductance (*g*_s_) and transpiration rate (*E*) of the plants were evaluated (Fig. 5). Under control conditions, no differences were found between WT and *Scdr2* plants. Drought stress reduced *A* in WT and *Scdr2* plants in a similar way, although, *Scdr2* plants had a higher *A* than WT plants in general (Fig. 5A). This trend was more evident under salinity conditions (Fig. 5C). In both stresses, *A* rates in *Scdr2* plants clearly showed a recovery trend after 3 d of re-watering, reaching about 50 % of the rates that were observed at the beginning of the experiment (Fig. 5B, C). In contrast, the recovery ability of WT plants was nearly zero. After 10 d of either drought or salt stress, *C*_i_ in *Scdr2* plants was sustained at about 300 μmol mol^–1^, while in WT plants, *C*_i_ was reduced to 200–250 μmol mol^–1^ (Fig. 5E, F). The stomatal conductance of the WT and *Scdr2* plants showed a similar behaviour to that observed for *A*, again being superior in the transgenic lines (Fig. 5H, I). *E* was reduced to nearly zero in the drought-stressed WT and *Scdr2* plants, and only the *Scdr2* plants showed recovery of *E* after re-watering (Fig. 5K). During salt stress, the *Scdr2* plants were able to maintain a higher *E* than the WT plants throughout the stress period (Fig. 5L). After 3 d of re-watering, *E* in the *Scdr2* plants was 2 mmol m^–2^ s^–1^, twice the value observed in the WT plants. The fluorescence was also measured to evaluate the photosystem II (PSII) response under stress and control conditions. We obtained values of around 0.85 ± 0.02 among our treatments, and no statistical difference was observed. Our results suggest that the improved resilience showed by *Scdr2* plants is associated with the maintenance of the physiological status under salinity and drought stress.

### *Scdr2* maintains water content and dry mass under drought and salinity stress

Water content and shoot dry mass were evaluated in *Scdr2* and WT plants after 10 d of stress followed by 3 d of re-watering (Table 1). During both salinity and drought stresses, water content was reduced by 9 % in WT plants, while in the plants overexpressing *Scdr2,* water content was only marginally reduced (Table 1). Shoot dry mass of WT plants was reduced by 46 % under drought stress and by 58 % under salinity conditions (Table 1). In contrast, *Scdr2* overexpression allowed the transgenic plants to sustain a higher shoot dry mass, with a reduction of only 15–26 % when grown under either drought or salinity stress. Compared with the WT, shoot dry mass of *Scdr2* plants was 60 % higher under drought and 65 % higher under salinity (Table 1).

**Table 1:** Water content (WC) and shoot dry mass (SDM) of WT and *Scdr2* transgenic plants

	Treatment					
	Control		Drought		Salinity	
Genotype	WC (%)	SDM (g)	WC (%)	SDM (g)	WC (%)	SDM (g)
WT	91.87 ± 1.84	0.27 ± 0.01	83.11 ± 1.99	0.15 ± 0.02	81.73 ± 2.71	0.11 ± 0.01
*Scdr2-1*	93.23 ± 0.18	0.29 ± 0.01	88.44 ± 0.21*	0.21 ± 0.03*	89.62 ± 0.29*	0.24 ± 0.02*
*Scdr2-2*	93.26 ± 1.30	0.28 ± 0.01	88.20 ± 0.35*	0.22 ± 0.06*	92.30 ± 0.74*	0.29 ± 0.02*
*Scdr2-3*	93.30 ± 0.66	0.29 ± 0.02	89.28 ± 1.41*	0.23 ± 0.05*	87.15 ± 1.83*	0.25 ± 0.02*

Thirty-day-old plants were exposed to 200 mm mannitol (drought) or 175 mm NaCl (salinity) for 10 d and then allowed to recover for 3 d with pure water. A parallel set of plants were maintained as controls and were irrigated with water only. Values represent the mean of three independent experiments. An asterisk indicates statistically significant differences compared with the WT plants in each treatment (*P* < 0.001, *n* = 5).

### *Scdr2* reduces oxidative stress damage

One of the initial consequences of abiotic stresses is oxidative damage (Bartels and Sunkar, 2005). Therefore, we evaluated H_2_O_2_ accumulation in the leaves of transgenic and WT plants. Interestingly, even under control conditions, the transgenic plants showed about 33 % lower levels of H_2_O_2_ than WT plants (Table 2). Following 10 d of drought stress and 3 d of re-watering, the H_2_O_2_ levels reached 180 nmol H_2_O_2_ g^–1^ f. wt in the WT plants, while the *Scdr2* transgenic plants were significantly less affected (*P* < 0.001), having levels of 125–140 nmol H_2_O_2_ g^–1^ f. wt. A similar pattern was observed in the salt-stressed plants: while WT plants produced 230 nmol H_2_O_2_ g^–1^ f. wt, *Scdr2* plants were significantly less affected (*P* < 0.001), having levels between 160 and 175 nmol H_2_O_2_ g^–1^ f. wt (Table 2). These results indicate that *Scdr2* plants had lower oxidative damage than WT plants, which would lead to less detrimental physiological responses, providing higher tolerance to water deficit and salt stress.

**Table 2:** Hydrogen peroxide accumulation in leaves of WT and *Scdr2* transgenic tobacco plants

	Treatment		
Genotype	Control	Drought	Salinity
WT	125.98 ± 0.34	165.32 ± 3.38	217.25 ± 7.57
*Scdr2-1*	106.28 ± 5.86*	131.51 ± 1.46*	162.61 ± 1.96*
*Scdr2-2*	108.23 ± 2.44*	140.19 ± 0.50*	158.21 ± 2.60*
*Scdr2-3*	114.31 ± 2.36*	149.04 ± 2.56*	162.44 ± 10.12*

Thirty-day-old plants were exposed for 10 d to 200 mm mannitol (drought) or 175 mm NaCl (salinity). The control plants were irrigated with pure water. H_2_O_2_ levels expressed in nmol H_2_O_2_ (g f. wt)^–1^ were determined in WT and *Scdr2* transgenic lines using Fe-Xylenol orange. The data are represented as the mean ± SD from three independent experiments (*n* = 5). An asterisk indicates statistically significant differences compared with controls (*P* < 0.001).

## DISCUSSION

Salinity and drought conditions trigger a diversity of responses, resulting in co-operative interaction among multiple physiological, biochemical and morphological changes. The identification and characterization of genes that are associated with these responses are crucial for developing new cultivars with improved tolerance, thus guaranteeing sustainable crop yields under challenging environmental conditions. Several sugarcane stress-induced genes that are putatively linked to drought and salinity stresses have been identified (Rocha *et al.*, 2007; Rodrigues *et al.*, 2011), but so far only one of them has been characterized (Begcy *et al.*, 2012). In this study, a novel drought-modulated small gene from sugarcane was functionally characterized. As described for rice, wheat, maize and sugarcane (Efeoglu *et al.*, 2009; Majlath *et al.*, 2012; Basu *et al.*, 2016; Campbell *et al.*, 2017; Ferreira *et al.*, 2017), drought stress promotes different responses between species and varieties. These types of responses were observed for the *Scdr2* gene in SP83-5073, SP90-1638, SP83-2847 and SP86-155, four commercial cultivars that differ in their degree of drought tolerance (Fig. 1). *Scdr2* gene expression was induced by drought in each cultivar, but there was no clear trend in the timing of the induction, nor a direct relationship with the different levels of drought tolerance in the four cultivars. Our data comparing the expression profiles of four different cultivars highlight that, even in the same species, gene expression profiles can show a wide array of responses. However, we could not exclude the possibility that the variation in gene expression might also be related to the different physiological stress experienced by each cultivar. This complex pattern was also observed when comparing the transcriptomes of two rice cultivars that differ in their tolerance to drought and salinity (Walia *et al.*, 2005; Lenka *et al.*, 2011). These data suggest that, besides the conserved responses to drought, other pathways involving several genes with a wide array of responses can also play key roles under environmental stresses. Although we are currently unable to explain the highly complex regulation of this gene in sugarcane, the *Scdr2* overexpression in transgenic tobacco plants strongly supports the hypothesis that *Scdr2* is involved in the sugarcane response to drought and salinity stresses.

The ScDR2 amino acid sequence (Supplementary Data Fig. S2) has a high degree of similarity to several proteins that have not yet been characterized (Supplementary Data Fig. S3). ScDR2 contains 16 % lysine and 8 % glutamate residues, as well as a conserved domain (small family of metazoan zinc-binding proteins – zf-met2) in the C-terminus. Members of the zf-met2 family are short proteins that have been suggested as potential regulators of abiotic and biotic stress responses (Kodaira *et al.*, 2011; Gupta *et al.*, 2012). Interestingly, other *Scdr2* homologues also encoded short proteins in the range of 74–79 amino acids and were found in monocotyledonous and dicotyledonous plants as well as in algae and fungi species (Supplementary Data Fig. S3A). Since *Scdr2* homologues were found in multiple species, including algae, fungi and plants, we suggest that this gene has an early evolutionary origin. In addition, because *Scdr2* is highly conserved throughout the kingdoms (Supplementary Data Fig. S3A), this conservation could indicate a conserved function. The protective role of *Scdr2* when overexpressed in tobacco plants is further evidence that its function has been conserved during evolution.

In general, seed germination and early seedling growth are among the developmental stages with the highest susceptibility to abiotic stresses in many crops (Li *et al.*, 2013; Debez *et al.*, 2018). Some environmental factors, including high temperatures (Liu *et al.*, 2015), water deficit (Sadeghian and Yavari, 2004), salinity (Debez *et al.*, 2018) and a combination of other stresses (Pandey *et al.*, 2017), can cause a significant delay in or even block seed germination. This effect was also observed in tobacco seeds that were exposed to high salinity levels and low water availability (Begcy *et al*., 2011, 2012). Poor seed germination may result in irregular establishment on soil, poor crop performance and reduced productivity (Sadeghian and Yavari, 2004; Li *et al.*, 2013; Liu *et al.*, 2015). A desirable plant characteristic to produce important commercial cultivars in field conditions is the ability of the seed to germinate rapidly and homogeneously under stress. The performance of the *Scdr2* tobacco seeds was better than that of the WT seeds in response to drought and salinity stresses, showing 35 % higher germination under severe drought (Fig. 3B) and 25 % under severe salt stress (Fig. 3E). Taken together, these results indicate that *Scdr2* plays a protective role during seed germination.

We have also observed that *Scdr2* overexpression positively affects younger plants submitted to environmental stresses. *Scdr2* transgenic lines showed a marked ability to recover from both drought and salinity stresses (Fig. 4). The ability to withstand these stresses was correlated with higher water levels in the leaves of the *Scdr2* plants (Table 1). The photosynthetic parameters of the *Scdr2* plants, including the rates of transpiration, net photosynthesis, stomatal conductance and internal leaf CO_2_ concentration, were less affected by salt or drought stress than were those of WT plants (Fig. 5). However, the photochemical efficiency of PSII was not affected in *Scdr2* and WT plants under water and salinity stress despite the observed changes in the photosynthetic rate (Fig. 5). Transgenic lines and WT plants maintained values of 0.85 ± 0.02, so the decrease of photosynthesis occurred due to decreased stomatal conductance rather than PSII damage. Since the overexpression of myo-inositol *O*-methyltransferase (IMT1) in *Nicotiana tabacum* and *Arabidopsis thaliana* provided protection against stress without changes into photochemical light-trapping reactions (Sheveleva *et al.*, 1997; Ahn *et al.*, 2011), we can conclude that *Scdr2* physiological improvement was related to photosynthetic (*A*, *Ci*, *g*_s_ and *E*), rather than photochemical parameters.

The highest stomatal conductance displayed by *Scdr2* plants might be partially explained by the interaction between ROS and ABA, since ABA induces stomatal closure mainly by provoking an efflux of potassium and some anions from the guard cells (Kim *et al.*, 2010) and the production of ROS, which activates Ca^2+^ influx channels in the plasma membrane (Murata *et al.*, 2001; Sierla *et al.*, 2016). ROS signalling is one of the primary responses to several abiotic stresses, including drought and salinity. Tobacco plants overexpressing the sugarcane *Scdr2* gene showed significantly lower levels of H_2_O_2_ compared with WT tobacco plants (Table 2). ROS production and scavenging under optimal growth conditions are maintained in an effective equilibrium, thus preserving a balance in cell homeostasis. However, under a variety of stress conditions, including drought and salinity, ROS generation increases drastically, exceeding the scavenging capacity and leading to an elevated ROS accumulation, causing progressive oxidative damage and ultimately cell death. Therefore, increased tolerance to environmental stresses has been linked to an adequate capacity to scavenge toxic levels of ROS (Zorov *et al.*, 2014; Petrov *et al.*, 2015). Thus, the enhanced tolerance to drought and salinity achieved by overexpressing the sugarcane *Scdr2* gene could be associated with the maintenance of an efficient anti-oxidative system, leading to adequate scavenging and detoxification of excessive ROS.

Our results show that the ability to overcome drought and salinity stresses due to *Scdr2* overexpression led to higher biomass accumulation (Table 1). Increased biomass accumulation under abiotic stress conditions is a key feature in coping with the challenges that are imposed in new areas of sugarcane production in Brazil. In the *Cerrados* (Brazil’s savannah), a potential area for sugarcane growth, environmental stresses are responsible for significant reductions in yield. Therefore, the production of sugarcane plants that overexpress genes conferring tolerance to drought will certainly be an interesting biotechnological approach. Our data shed light on the role of a sugarcane gene that responds to drought and salinity. Because *Scdr2* does not encode a protein with a known function or any other type of signature, further work will be necessary to formulate a hypothesis regarding the mode of action of this gene. Our work highlights the relevance of a group of genes that encode unknown proteins, which make up an important fraction in most genome studies.

## SUPPLEMENTARY DATA

Supplementary data are available online at https://academic.oup.com/aob and consist of the following. Figure S1: expression profile of *Scdr2* homologues in two maize inbred lines under drought as reported by Zheng *et al.* (2010). Figure S2: (A) DNA and predicted signal peptide and (B) deduced protein sequence of the *Scdr2* gene. Figure S3: protein sequence alignment of sugarcane ScDR2 withits homologues.

mcz044_suppl_Figure_Caption

mcz044_suppl_Figure_1

mcz044_suppl_Figure_2

mcz044_suppl_Figure_3

## FUNDING

K.B. was the recipient of a CNPq (National Council for Scientific and Technological Development) fellowship. This work was developed as part of the research network from the National Institute of Science and Technology of Bioethanol (Ministry of Science and Technology and grant 2008/57908-6 from FAPESP), and it was also partially funded by grant 815/07 from FINEP (Research and Projects Financing).
