# Supplementary material for: Overexpression of an evolutionarily conserved drought-responsive sugarcane gene enhances salinity and drought resilience
Source: Ann Bot. 2019 May 24;124(4):691–700. doi: 10.1093/aob/mcz044 (PMC6821327; doi:10.1093/aob/mcz044)
Supplement: mcz044_suppl_Figure_Caption [file mcz044_suppl_figure_caption.docx]

**Supplementary data figure captions**

**Fig S1:** Expression profile of *Scdr2* homologous in two maize inbred lines under drought as reported by Zheng et al. ([2010](#_ENREF_48)). Raw data available in the Gene Expression Omnibus (GEO), series GSE16567.

**Fig. S2**: **A.** DNA and predicted signal peptide (in yellow) and **B.** deduced protein sequence of the *Scdr2* gene (Accession number: AFY12046). The sequences were obtained from the SUCEST database corresponding to the SAS (Sugarcane Assembled Sequence) SCRFLR2038D12.g.

**Fig. S3:** Protein sequence alignment of sugarcane ScDR2 (SAS SCRFLR2038D12.g) with homologues
