# Supplementary figures and images for: Overexpression of an evolutionarily conserved drought-responsive sugarcane gene enhances salinity and drought resilience
Source: Ann Bot. 2019 May 24;124(4):691–700. doi: 10.1093/aob/mcz044 (PMC6821327; doi:10.1093/aob/mcz044)

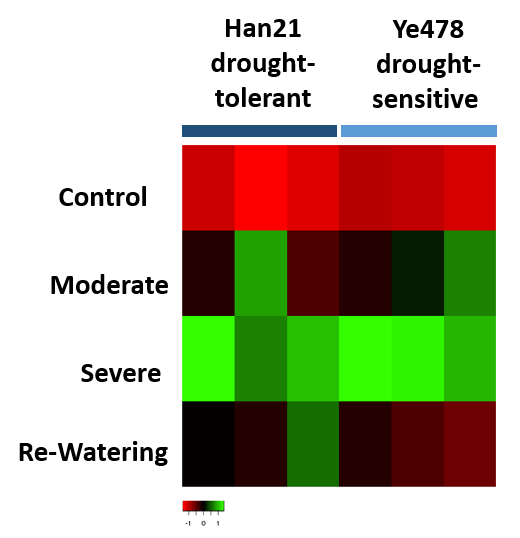

Supplement: mcz044_suppl_Figure_1 [file mcz044_suppl_figure_1.png]
