# Supplementary material for: Overexpression of an evolutionarily conserved drought-responsive sugarcane gene enhances salinity and drought resilience
Source: Ann Bot. 2019 May 24;124(4):691–700. doi: 10.1093/aob/mcz044 (PMC6821327; doi:10.1093/aob/mcz044)
Supplement: mcz044_suppl_Figure_2 [file mcz044_suppl_figure_2.docx]

**
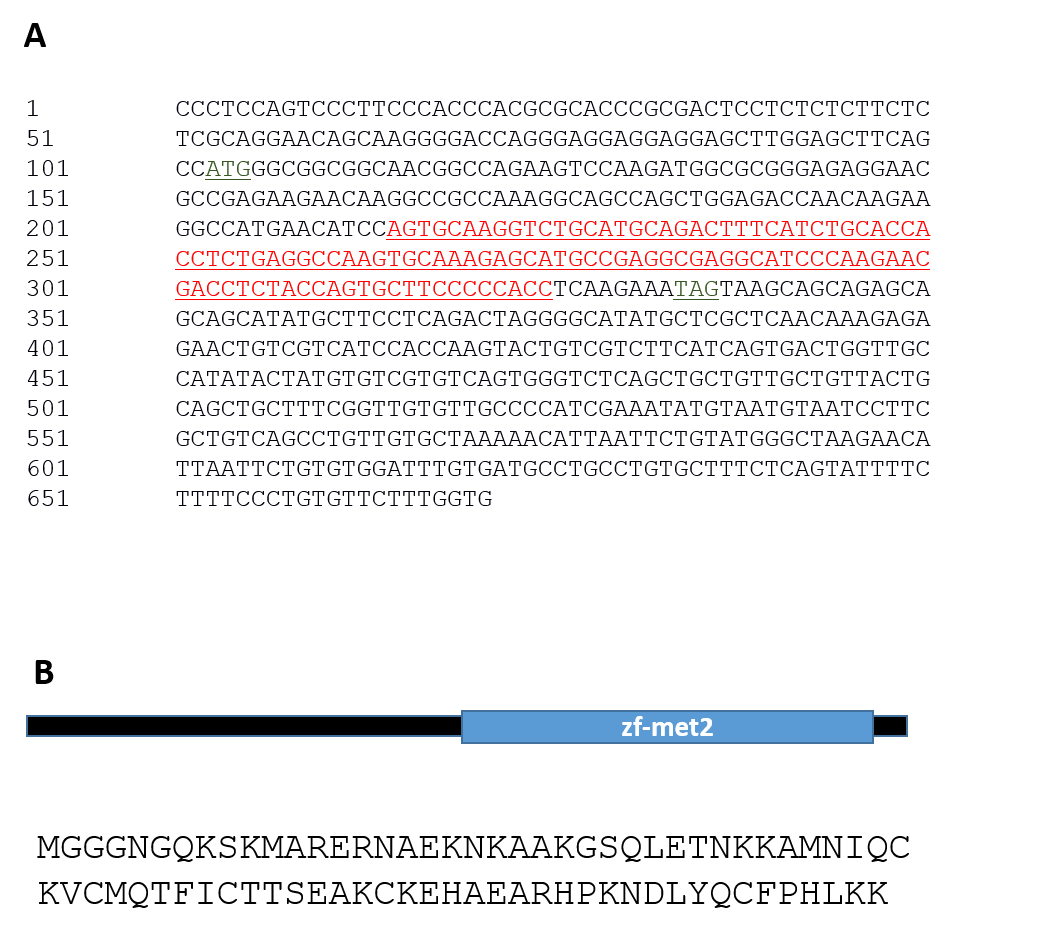
**

**Fig. S2**. **A.** DNA and predicted signal peptide (in yellow) and **B.** deduced protein sequence of the *Scdr2* gene (Accession number: AFY12046). The sequences were obtained from the SUCEST database corresponding to the SAS (Sugarcane Assembled Sequence) SCRFLR2038D12.g.
