# Supplementary material for: Overexpression of an evolutionarily conserved drought-responsive sugarcane gene enhances salinity and drought resilience
Source: Ann Bot. 2019 May 24;124(4):691–700. doi: 10.1093/aob/mcz044 (PMC6821327; doi:10.1093/aob/mcz044)
Supplement: mcz044_suppl_Figure_3 [file mcz044_suppl_figure_3.docx]

**
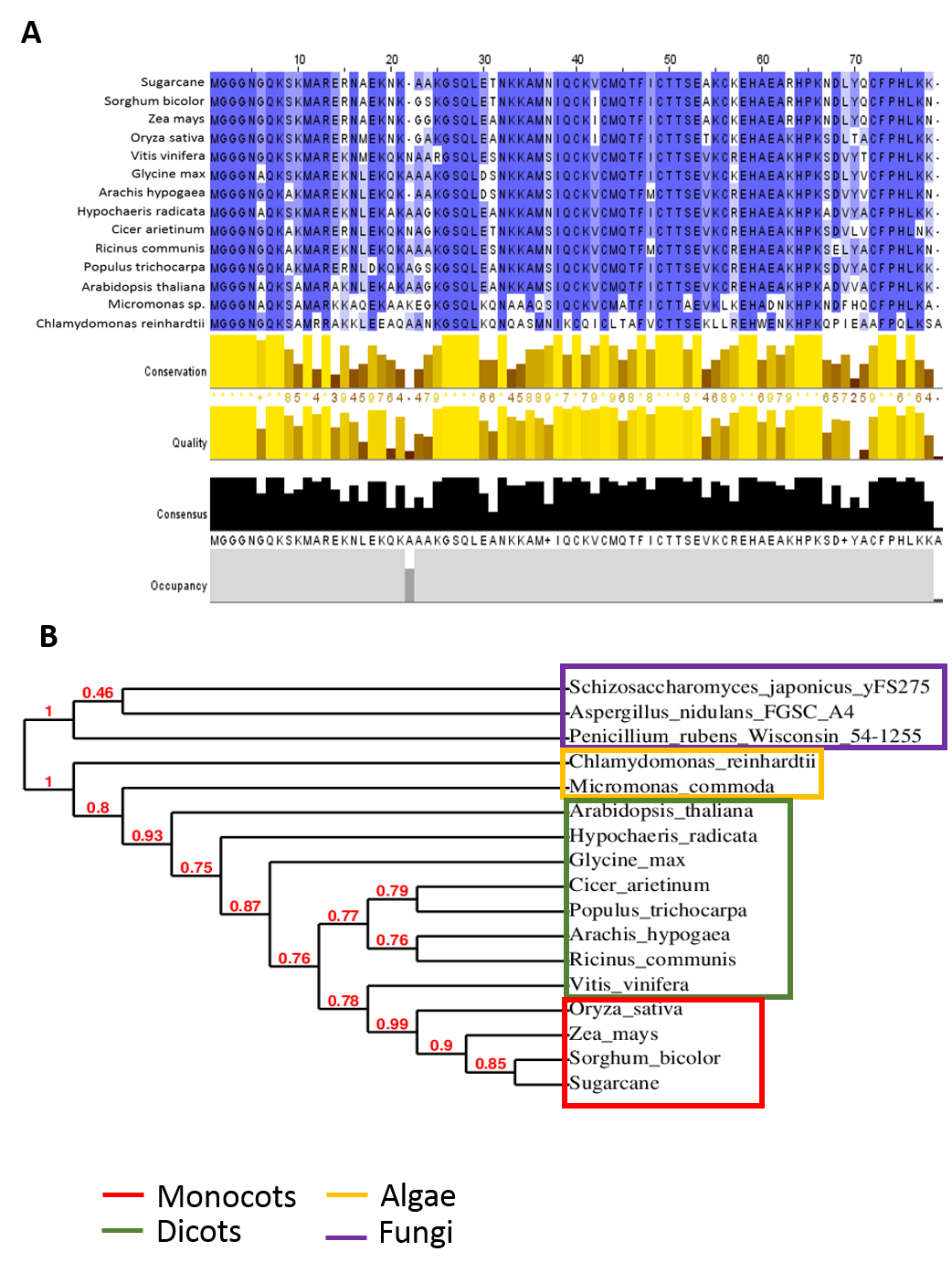
**

**Fig. S3.** Protein sequence alignment of sugarcane ScDR2 (SAS SCRFLR2038D12.g) with homologues from several species. **A.** Alignment with other plant ScDR2 proteins. **B.** Neighbor-joining tree of the *Sc*DR2 protein sequences from sugarcane and other organisms. All the sequences were aligned using the Clustal2W software. Sequences accession numbers: Sugarcane (AFY12046), *Sorghum bicolor* (XP_002459234.1), *Zea mays* (ACG30543.1), *Oryza sativa* (NP_001058751.1), *Vitis vinifera* (XP_002285206.1), *Glycine max* (ACU14952.1), *Arachis hypogaea* (ACF74320.1), *Hypochaeris radicata* (BAH97744.1), *Cicer arietinum* (CAA10129.1), *Ricinus communis* (XP_002533162.1), *Populus trichocarpa* (XP_002302417.1), *Arabidopsis thaliana* (NP_565547.1), *Micromonas sp.* (XP_002508158.1), *Chlamydomonas reinhardtii* (XP_001689924), *Penicillium rubens* (XP_002565772), *Aspergillus nidulans* (XP_680886), and *Schizosaccharomyces japonicus* (XP_002175475). Strong blue color indicates high degree of residue conservation. Light blue indicates low degree of residue conservation.
